# A possible precursor prior to the Lushan earthquake from GPS observations in the southern Longmenshan

**DOI:** 10.1038/s41598-020-77634-6

**Published:** 2020-11-30

**Authors:** Qixin Wang, Xiwei Xu, Zaisen Jiang, John Suppe

**Affiliations:** 1 National Institute of Natural Hazards, MEMC, Beijing, 100085 China; 2 Institute of Earthquake Forecasting, China Earthquake Administration, Beijing, 100036 China; 3 Department of Earth and Atmospheric Sciences, University of Houston, Houston, TX USA

**Keywords:** Geodynamics, Tectonics

## Abstract

Global Positioning System (GPS) stations installed in and around the epicenter of the Lushan earthquake (Mw 6.7), which occurred almost 5 years after the 2008 Wenchuan earthquake, recorded preseismic deformation corresponding to the Lushan earthquake within the southern Longmenshan thrust belt. A half-space dislocation model is used to simulate the theoretical values of the postseismic displacements caused by the 2008 Wenchuan earthquake, and after transforming the reference frame and filtering the GPS displacement time series, the theoretical and observed GPS values are compared to identify the geodetic anomaly preceding the Lushan earthquake. The abnormal extent of this geodetic anomaly decreases with increasing epicentral distance for each GPS site. This geodetic signal reflects preslip along a locked section of the 2013 seismogenic fault, which caused the accumulation of elastic strain energy until the faulting strength was overcome, thereby generating the Lushan earthquake. Hence, this anomaly might be used as an observable and identifiable precursor to forecast an impending earthquake within a period of less than two and half years before its occurrence.

## Introduction

Predicting the magnitude and location of an earthquake prior to an impending seismic event has been a controversial subject of research over the past two decades^1–3^. At present, earthquake forecasting is a very difficult issue because very little about the earthquake mechanism is understood; moreover, the instruments needed to observe an earthquake are often not situated sufficiently close to the epicenter at the correct time^4–6^. Since the Global Positioning System (GPS) technology, some researchers have tried to detect possible precursors before an earthquake by analyzing preseismic strain rate^7^, baseline distance^8^ or ionospheric maps^9^. However, researchers have difficulty explaining the relationship between the observed precursors and the epicenters. Furthermore, the location of the epicenter cannot be forecasted before an earthquake. Consequently, whether an observable and identifiable precursor anomaly precedes an impending earthquake has yet to be resolved^10,11^.

**Figure 1:**
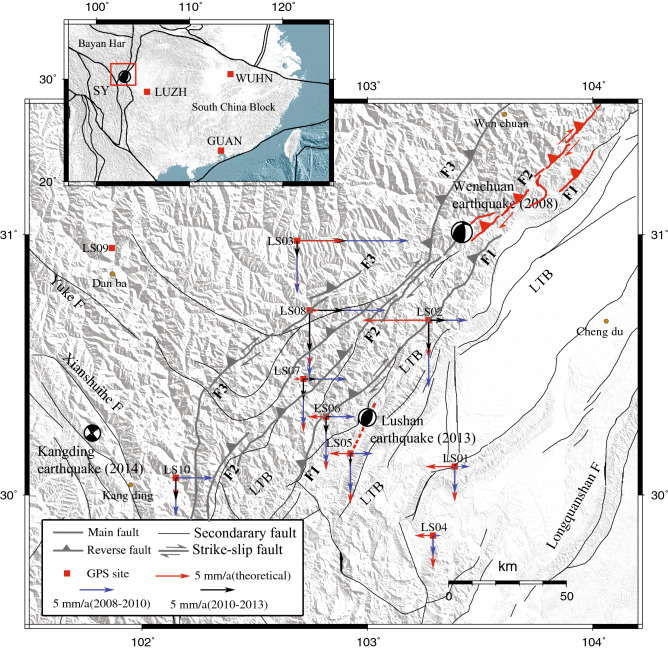
Sketch map showing the distribution of the installed continuous GPS stations (LS01–LS10) and the epicenters of the Lushan and Wenchuan earthquakes. WUHN, GUAN and LUZH in the inset map in the upper-left corner are the GPS reference stations in the South China Block, a stable tectonic domain. The red arrows are the postseismic theoretical velocities associated with the Wenchuan earthquake obtained by a half-space dislocation model. Blue arrows and black arrows are the observations at each GPS site. The LTB represents the Longmenshan thrust belt, which consists of the Pengguan fault (F1), Beichuan fault (F2) and Maowen fault (F3). The SY represents the Sichuan-Yunnan Block. Lines with solid triangles and arrows represent reverse faults and strike-slip faults, respectively. The red solid lines represent the surface rupture zone of the Wenchuan earthquake, while the red dashed lines represent the surface projection of the inferred seismogenic fault of the Lushan earthquake. Map is generated by GMT software, v. 4.5.15 (http://www.soest.hawaii.edu/gmt/).

The Longmenshan thrust belt (LTB) is a N42° ± 5°E-trending crustal shortening boundary between the Bayan Har Block and the South China Block along the eastern margin of the Qinghai-Tibet Plateau^12,13^. The LTB has attracted considerable attention in the last 10 years, particularly as a result of the 2008 Mw 7.9 Wenchuan and 2013 Mw 6.7 Lushan earthquakes that occurred along the thrust boundary^14–19^. The Wenchuan earthquake produced a complicated 240-km-long surface rupture zone in the middle segment of the LTB, while the Lushan earthquake occurred along the southern segment as a blind reverse-faulting event^14–17,20–22^. The distance between the rupture zones of these two earthquakes is 90 km^23,24^, and few earthquakes have occurred within this gap. During the Wenchuan earthquake, a significant stress increase resulted from coseismic oblique slip, which had the potential to trigger or hasten the occurrence of a destructive earthquake on the southern segment of the LTB^18,19^. Therefore, a well-designed temporary continuous GPS network comprising 10 stations (Fig. 1) was installed in and around the southern segment of the LTB soon after the Wenchuan earthquake by the Institute of Earthquake Forecasting, China Earthquake Administration (Fig. 1; Supplementary Table S1). All of these GPS stations recorded the preseismic deformation corresponding to the Lushan earthquake. Accordingly, any identifiable precursory signals recorded in the vicinity of the epicenter may be detected, thereby revealing heretofore unreported aspects of the earthquake process, and this knowledge may be used to forecast destructive earthquakes in and around the eastern Tibetan Plateau.

## Results

### Observations and simulations

The results reveal that the theoretical postseismic displacements at stations LS01, LS02, LS04, LS05, LS06, LS07 and LS10 have a westward component (Fig. 1). As previous work has shown^14^, the Wenchuan earthquake was a thrust fault earthquake. Thus, the westward component is reasonable because most of these stations, except LS07, are located on the footwall of the Beichuan fault (F2) (Fig. 1). LS07 is located far from the source fault of the earthquake, but the normal distance between the station and the fault is small. Therefore, it is difficult to judge the direction of the postseismic displacement based on its location^25^. We can only estimate the direction with theoretical value. However, in the reference framework of the South China Block, all of the GPS stations have an eastward component because the observations also include the eastward secular motion, which is larger than the westward postseismic slip due to the Wenchuan earthquake and thus controls the observed displacements at all the GPS stations. Motions of both the observed and the theoretical values have the same southward direction (Fig. 1).

### Characteristics of observed time series

**Figure 2:**
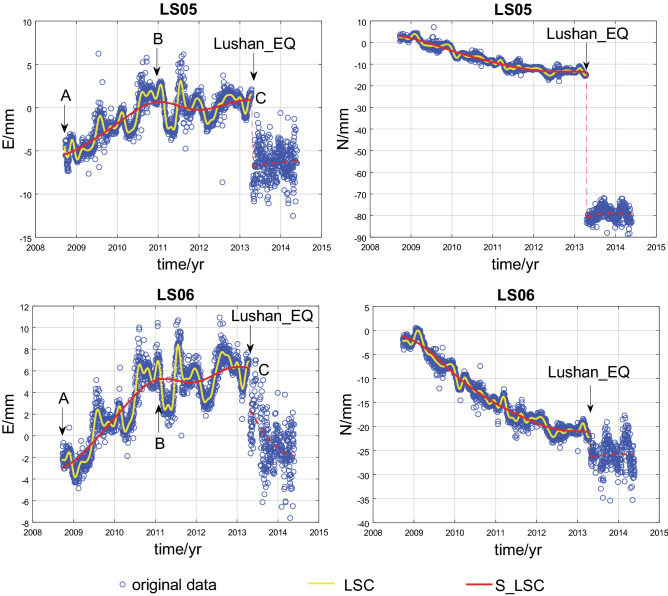
(**a**) Eastward and (**b**) northward displacement time series of stations LS05 and LS06. Blue circles are the original data; the yellow line represents the data filtered by the least-squares collocation method; and the red line signifies the secondary data without periodicity filtered by the least-squares collocation method. Section AB presents a linearly increasing trend and a locked seismogenic fault. Beyond point B, section BC shows a stable stage and preslip motion along the seismogenic fault before the Lushan earthquake. Map is generated by Matlab software, v. R2018a (https://www.mathworks.com/).

**Figure 3:**
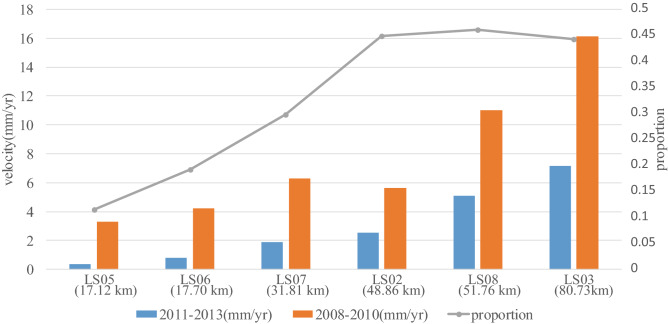
Eastward velocities during 2011–2013 and 2008–2010 and a comparison between the two periods. The abscissa is the name of the station and its epicentral distance. The bar chart displays the velocity of each station in the Bayan Har Block except LS10. The gray line is the difference between the velocity during 2011–2013 and that during 2008–2010. Map is generated by Matlab software, v. R2018a (https://www.mathworks.com/).

The site closest to the epicenter of the Lushan earthquake is station LS05 on the Bayan Har Block (approximately 17 km), followed by station LS06 (Supplementary Table S1). As shown above, the eastward components at both stations contain both the secular motion and the theoretical postslip component associated with the Wenchuan earthquake. Theoretically, the eastward secular motion of the Bayan Har Block remains constant as a long-term loading strain, while the westward theoretical postslip component gradually decays to zero as a short-term loading strain, suggesting that the eastward displacements of these two stations should theoretically increase with time. We do observe a linear stress-induced increase in the eastward displacements of both LS05 and LS06 with velocities of 3.3 ~ 4.2 mm/yr in the first 2 years after installation in addition to a similar pattern in the displacement time series (Figs. 2 and 3). However, the velocities at these stations obviously decrease after 2011 to very low values of 0.3 ~ 0.8 mm/yr (Figs. 2 and 3). As a result, LS05 remained stable, exhibiting almost no eastward motion there after 2011(Fig. 2). This finding demonstrates that the beginning of 2011 marks a temporal inflection point, after which little or no near-field eastward displacement occurred at or around the epicenter, as depicted in section BC of the eastward displacement time series of stations LS05 and LS06 (Fig. 2).

Similarly, the eastward components of stations LS02 and LS07, approximately 49 km and 32 km from the epicenter, respectively (Supplementary Table S1), also resulted mainly from secular motion after subtracting the postslip deformation due to the Wenchuan earthquake. We also observe a preseismic anomaly similar to that reflected by the eastward displacement time series in the GPS data from LS05 and LS06 (Fig. 2), but this anomaly is not as obvious as that at LS05 and LS06. The velocity variations at LS02 and LS07 also display a temporal inflection point at the beginning of 2011, after which the eastward velocities diminish (Supplementary Fig. S1), slowing obviously from 5.6–6.3 mm/yr generally before 2011 to 1.9–2.5 mm/yr after 2011 until the occurrence of the Lushan earthquake (Supplementary Fig. S1).

The data from stations LS03 and LS08, whose epicentral distances are greater than 50 km (Fig. 1 and Supplementary Table S1), are quite different from those at stations LS05 and LS06. The secular motion and postslip deformation caused by the Wenchuan earthquake at LS03 and LS08 have the same eastward components. Moreover, both their northward and eastward components readily conform to the postseismic exponential model^21^ without obvious anomalies, and the velocities of the eastward displacements gradually decrease during the observation period, but no temporal inflection points are observed in the eastward displacement time series of LS03 and LS08 (Supplementary Fig. S2).

Station LS10 is the GPS site farthest from the epicenter of the Lushan earthquake (Supplementary Table S1), and the velocity at this site decays after February 2011 (Supplementary Fig. S1). However, the anomaly at LS10 may not be related to the Lushan earthquake owing to its location on the eastern side of the Xianshuihe fault, a major left-lateral strike-slip fault^26^. In contrast, this anomaly may be related to the 2014 Mw 5.9 Kangding earthquake^27^ (Fig. 1). Furthermore, stations LS01 and LS04 are located on the footwall of the LTB on the South China Block, which is more rigid than the Bayan Har Block^28^. After transforming the reference framework to the South China Block, the motions at LS01 and LS04 are steady, and their eastward and northward motions both remain stable before the Lushan earthquake (Supplementary Fig. S3).

### The relationship between the anomaly and the epicentral distance

The above analysis reveals an obvious decrease in the eastward velocity surrounding the epicenter of the Lushan earthquake approximately two and half years before its occurrence. In addition, the eastward velocity at each station is increasingly reduced with increasing proximity to the epicenter. To determine the relationship between the anomaly and the epicentral distance, the velocities recorded during 2011–2013 are compared with those recorded during 2008–2010 in the Bayan Har Block. The results demonstrate that stations within 50 km of the epicenter present obviously reduced eastward velocities before the Lushan earthquake and that the reduction in the eastward velocity is inversely proportional to the epicentral distance (Fig. 3). The velocity gradually increases outward for the stations within a range of ~ 50 km, whereas the velocities for the remaining stations outside the ~ 50 km radius exhibit a similar pattern. Thus, the dramatic decrease in the eastward velocity (even to zero) at the epicenter observed at these continuous GPS stations occurred only within a radius of ~ 50 km centered at the expected epicenter. This near-field phenomenon may be used as a precursor to locate impending earthquakes.

## Discussion

### Ultimate strength of the fault

The LTB has suffered from oblique crustal shortening throughout the Quaternary owing to the east-southeastward motion of the Bayan Har Block and its collision with the South China Block, which has accommodated India’s northward penetration into Eurasia^12,13^. When this oblique shortening strain accumulates sufficiently to reach the ultimate strength of a locked fault segment within the LTB, that fault segment fails and leads to unstable faulting, that is, an earthquake. The slopes of the eastward displacement time series at stations LS05 and LS06 change significantly at the inflection point (B) (Fig. 2). Before point B, the seismogenic fault should be locked; however, the linear increase in strain at a rate larger than 3.3 mm/yr in the eastward direction demonstrates that elastic strain continues to accumulate. After point B, approximately two and a half years before the Lushan earthquake, the strain seems to reach its peak and remains constant for stations LS05 and LS06 (Fig. 2) or accumulates at a lower rate at stations LS02 and LS07 (Supplementary Fig. S1); consequently, seismic energy is ready to be released on the seismogenic fault.

### Seismic nucleation

**Figure 4:**
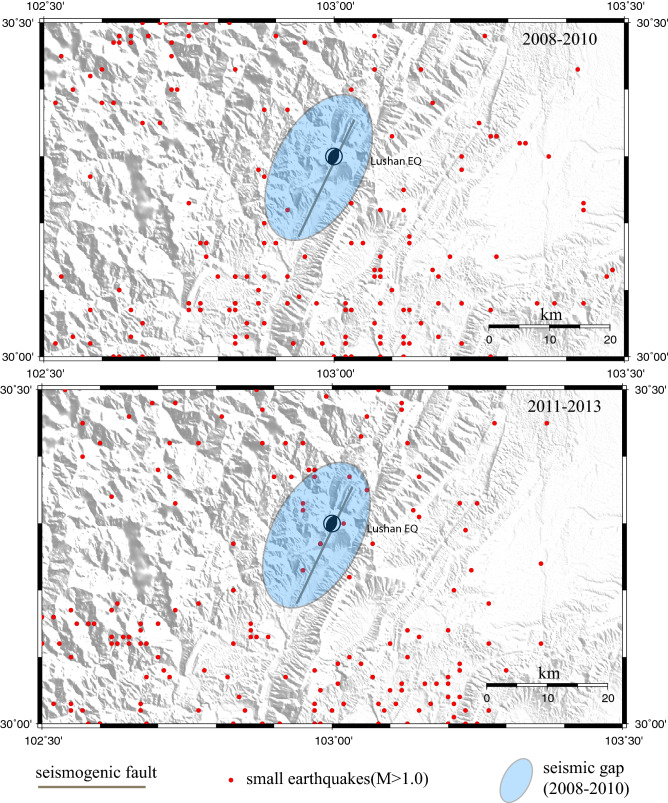
Small earthquakes in the vicinity of the southern segment of the LTB in the 2008–2010 and 2011–2013 periods. Red circles are small earthquake locations; the gray solid line is the seismogenic fault of the Lushan earthquake as mentioned in Fig. 1. Map is generated by GMT software, v. 4.5.15 (http://www.soest.hawaii.edu/gmt/).

Section BC in the eastward displacement time series coincides well with the almost aseismic preslip and upward-cascading process in the laboratory that initiates a relatively large dynamic rupture on the seismogenic fault^29–32^. The seismicity recorded by the Sichuan Seismic Network confirms that the seismic activity at the epicenter is weak, but more seismicity occurred in the epicenter and its adjacent areas during the period from 2011 to 2013 before the Lushan earthquake than during the period from 2008 to 2010 (Fig. 4). This implies that the preslip (nucleation) phase is aseismic and weakens the seismogenic fault^28–31^. Thus, the characteristics of the eastward displacement time series in section BC on approximately two and half years before the Lushan earthquake may be taken as a possible precursor for earthquake forecasting.

### Seismic gap of the LTB

Among these stations, LS02 is special, as it is located in the seismic gap between the Lushan and Wenchuan ruptures on the LTB^14,15,24^, and the station is far from the epicenter of the Lushan earthquake. Moreover, stations LS02 and LS05 are both located near the southern segment of the Pengguan fault that did not rupture during the Wenchuan earthquake. However, the accumulated strain at LS05 reached its peak, and an anomaly involving eastward displacement occurred before the Lushan earthquake; this is in contrast to LS02, which still recorded an eastward displacement. To a certain extent, this may explain why the earthquake occurred near station LS05 rather than near station LS02 and why the fault segment between the Lushan and Wenchuan earthquakes did not rupture.

### Reductions in eastward velocity

As shown in Fig. 2 and Supplementary Figs. S1, significant reductions in velocity are observed only for the eastward displacements for the near-field stations. This can be explained by the regional tectonic background motion within the LTB and its adjacent areas. Both geodetic observations and tectonic research have verified that the regional principal compressive stresses are oriented nearly E-W^33–35^. Thus, before the Lushan earthquake, the strain accumulation rate in the E-W direction was greater than that in the N-S direction. Another reason why we focus predominantly on the eastward component is that only three reference stations can be used to control the regional reference frame (Fig. 1). The eastward displacement of the South China Block calculated from these three stations is sufficiently stable to ensure the reliability of the results, whereas the northward displacement exhibits fluctuation (Supplementary Fig. S2).

## Data and methods

GPS data were collected from the temporary continuous GPS network. Nine of the stations have been operating since 2008, whereas station LS09 was installed in 2010 at a site more than 130 km northwest of the epicenter of the Lushan earthquake. Thus, in our research, we analyzed GPS data from the 9 sites (other than LS09). We obtained the preseismic displacement time series data from the 2005 International Terrestrial Reference System (ITRF2005) that were processed by GAMIT/GLOBK. Then, we transform the observations into the South China Block framework and filter time series with stacking and the least-squares collocation method (see the Supplementary).

The Lushan earthquake occurred on the southern segment of the LTB almost 5 years after the Wenchuan earthquake, and its epicenter is located within the domain of the newly installed continuous GPS network (Fig. 1). Since most of these GPS stations were affected by the Wenchuan earthquake, the postseismic displacement of the earthquake at each GPS station is simulated (see the Supplementary) by using a half-space dislocation model^36^. A Maxwell body and a layered structure model (see the Supplementary Materials) are used in the simulation.

## Supplementary information

Supplementary Information.

Supplementary Figure 1.

Supplementary Figure 2.

Supplementary Figure 3.

Supplementary Figure 4.

## Data Availability

The data that support the findings of this study are available from the Institute of Earthquake Forecasting, China Earthquake Administration, but restrictions apply to the availability of these data, which were used under license for the current study, and so are not publicly available. Data are however available from the first author (Email: qixin0321@163.com) upon reasonable request and with permission of the Institute of Earthquake Forecasting, China Earthquake Administration.
