# Supplementary material for: A possible precursor prior to the Lushan earthquake from GPS observations in the southern Longmenshan
Source: Sci Rep. 2020 Nov 30;10:20833. doi: 10.1038/s41598-020-77634-6 (PMC7704651; doi:10.1038/s41598-020-77634-6)
Supplement: Supplementary file 1 — Supplementary Information. [file 41598_2020_77634_MOESM1_ESM.docx]

**A possible precursor prior to the Lushan earthquake from GPS observations in the southern Longmenshan**

Qixin Wang^1^, Xiwei Xu^1*^, Zaisen Jiang^2^, John Suppe^3^

1. National Institute of Natural Hazards, MEMC, Beijing 100085, China

2. Institute of Earthquake Forecasting, China Earthquake Administration, Beijing 100036, China

3. Department of Earth and Atmospheric Sciences, University of Houston, Houston, Texas, USA

Supplementary Materials:

**Data processing and method**

After the Wenchuan earthquake, the results of inverting the rupture process, the coseismic slip difference among fault segments and the accumulation of deformation in the southern segment of the LTB showed that the southern segment was still locked and had a risk of producing a strong earthquake^1^. Based on this consideration, a temporary continuous Global Positioning System (GPS) network was deployed around the southern segment of the LTB by the Institute of Earthquake Forecasting, China Earthquake Administration, to investigate regional displacements and deformation. Most stations recorded preseismic displacements from 2008 to 2013 before the Lushan earthquake. We obtained these preseismic displacement time series data in the 2005 International Terrestrial Reference System (ITRF2005) that were processed by GAMIT/GLOBK^1,2^.

In the global reference frame, mainland China moves so intensely toward the east that crustal movement information cannot be significant; in addition, preseismic displacement anomalies may be obscured. Furthermore, the Lushan earthquake was caused by the collision of the Bayan Har Block (BYH) and South China Block (SC). Hence, any possible precursory signals would be observable in the relative motion between these two blocks, and the detection of these precursors constitutes the focus of our research. The Lushan earthquake occurred along the boundary of the SC, which behaves rigidly and moves stably^28^. To obtain the relative motion between the BYH and SC, we transform the displacement time series of 9 stations in the ITRF2005 reference frame into displacements in the SC reference frame; this transformation not only highlights crustal movement information but also reduces common mode errors to some extent^3^.

In this research, we use three sites (GUAN, LUZH, and WUHN) in the SC to compose the regional reference frame (Fig. 1) and transform the displacements recorded at 9 sites into displacements in the SC reference frame. The three reference stations are all located far from the epicenter of the Wenchuan earthquake and have little influence from the earthquake.

| $\left[ \begin{matrix} U_{e} \\ U_{n} \end{matrix} \right]=R\left[ \begin{matrix} \begin{matrix} -sin\phi cos\lambda& -sin\phi sin\lambda& cos\phi\end{matrix} \\ \begin{matrix} sin\lambda& -cos\lambda& 0 \end{matrix} \end{matrix} \right]\left[ \begin{matrix} \omega_{x} \\ \omega_{y} \\ \omega_{z} \end{matrix} \right]$ | (1) |
| --- | --- |

Eq. 1 shows the relationship between the displacement and Euler vector^4,5^, where $\mathbf{U}$ is the displacement and $\boldsymbol{\omega}$ is the Euler vector. In addition, $R$ is the Earth’s radius, and $\phi$and$\lambda$ are the latitude and longitude of the station, respectively. With Eq. 1, we can use the 3 reference stations to calculate the Euler vector of the SC. Then, we can determine the theoretical displacement of each site with the Euler vector and Eq. 1. After subtracting the theoretical value (Supplementary Fig. S2), we obtain the displacement of each GPS station in the SC reference frame. When we transformed the local framework, the eastward displacement was adequately stable to ensure the reliability of these findings; in contrast, the northward displacement exhibited some fluctuation (Supplementary Fig. S1). Therefore, we discuss only the eastward motion anomaly for each GPS station.

The common mode error (CME) is a major spatially correlated source of error in the continuous GPS solution and is mitigated through a technique commonly referred to as regional filtering^6,7^. To reduce the influence of the CME, we use the stacking method^7^ to filter the time series (Fig. 2 and Supplementary Fig. S1).

As shown in Fig. 2 and Supplementary Fig. S1, after transforming the reference frame, the original displacements (blue circles) contain higher-frequency errors and greater periodicity, while naturally occurring processes often remain at low frequencies^8^. To investigate the crustal movement more clearly, we use the least-squares collocation method^8^ (LSCM) to filter out higher frequencies and retain lower frequencies. The LSCM is robust and features fewer edge effects and greater stability than other filtering techniques. The basic equation of the LSCM is

| $V_{0}=A\Omega+UV_{s}+n$ | (2) |
| --- | --- |

where $V_{0}$ is the observed time series from GPS stations, $\Omega$ represents the long-term linear parameter, $V_{s}$ signifies information about deviations from linear characteristics, and $n$ is random errors^8^. If the time series data deviate from the long-term linear characteristic ($A\Omega$) but the deviated information ($V_{s}$) does not exceed the tolerance of the random error ($n$), significant crustal deformation is not observed. In contrast, we focus on the more significant deviations in this study. Based on the LSCM, the estimated values of $\Omega$ and $V_{s}$ are

| $\hat{\Omega}={(A^{T}{(C_{oo}+C_{nn})}^{-1}A)}^{-1}A^{T}{(C_{oo}+C_{nn})}^{-1}V_{0}$ | | (3) |
| --- | --- | --- |
| $\hat{V}_{s}=C_{uo}{(C_{oo}+C_{nn})}^{-1}(V_{0}-A\hat{\Omega})$ | (4) | |

where $C_{\mathrm{oo}}$ is the covariance of $V_{0}$, $C_{\mathrm{nn}}$ is the covariance matrix of observed errors, and $C_{\mathrm{uo}}$ represents the covariance matrix of all signals (including linear and nonlinear data). In Eq. 3 and Eq. 4, $C_{\mathrm{oo}}$ and $C_{\mathrm{uo}}$ are two unknown parameters that can be inferred from the following Gaussian covariance function (detailed discussions is provided by Jiang and Liu^9^).

| $F\left( d \right)=Aexp(-k^{2}d^{2})$ | (5) |
| --- | --- |

Eq. 5 implies the relation between the time interval $d$ and covariance $F\left( d \right)$. $A$ and $k$ are two unknown parameters to be obtained. With the observed time series, we can fit Eq. 4 and obtain $A$ and $k$. Then, $C_{oo}$ and $C_{uo}$ can be calculated with the known relation (Eq. 5). In this study, we first filter most random errors (black line) and then filter the periodicity (red line) with the LSCM (Fig. 2).

The Lushan earthquake occurred on the southern segment of the LTB nearly five years after the Wenchuan earthquake. The observations from the 9 GPS stations were influenced by the postseismic displacements caused by the Wenchuan earthquake. As previous works have shown, viscoelastic relaxation and afterslip are two major mechanisms of deformation after an earthquake. Because the directions of deformation from these two mechanisms are similar and because we analyze only the direction of postseismic deformation in this study, we simulate only the viscoelastic relaxation following the Wenchuan earthquake. With this consideration, we use PSGRN/PSCMP^35^ to simulate the postseismic displacements of these stations. Based on a multilayered viscoelastic half-space model, PSGRN/PSCMP software can simulate the coseismic and postseismic deformations of a certain earthquake. Most of our stations are located in the BYH, so we use the layered structure of the block described in Supplementary Fig. S4 and Table S2^10^. The input coseismic slip distribution is taken from the results of reference^16^, and a Maxwell body is used in the simulation. The software can simulate the cumulative displacement at each site after the Wenchuan earthquake. In our research, we extract the cumulative deformation between the time of the starting observation and the occurrence of the Lushan earthquake.

Because of the direction of the principal compressive stress and the fluctuation in the northward theoretical displacement (Supplementary Fig. S1), we discuss only the eastward displacement anomaly. We also calculate the displacement time series in the directions parallel and orthogonal to the seismogenic fault of the Lushan earthquake (Supplementary Fig. S3). However, because we use both eastward and northward displacements to obtain them, these results are not sufficiently persuasive.

**Tables**

Table S1 Information of GPS stations

| Name | Latitude(deg) | Longitude(deg) | E-Distance(km) | Time period(year) |
| --- | --- | --- | --- | --- |
| LS01 | 30.11 | 103.39 | 42.82 | 2008.69-2013.3 |
| LS02 | 30.67 | 103.27 | 48.86 | 2008.70-2013.3 |
| LS03 | 30.98 | 102.69 | 80.73 | 2008.72-2013.3 |
| LS04 | 29.84 | 103.29 | 57.90 | 2008.69-2013.3 |
| LS05 | 30.16 | 102.92 | 17.12 | 2008.69-2014.3 |
| LS06 | 30.30 | 102.82 | 17.70 | 2008.71-2014.3 |
| LS07 | 30.45 | 102.72 | 31.81 | 2008.69-2013.3 |
| LS08 | 30.71 | 102.74 | 51.76 | 2008.69-2013.3 |
| LS09 | 30.95 | 101.87 | 130.39 | / |
| LS10 | 30.07 | 102.15 | 85.77 | 2008.73-2013.3 |

E-Distance: epicentral distance of each station

Table S2 Layered structure of Bayan Har Block^10^

| Material | Layer | Thickness/km | $v_{p}/km\cdot s^{-1}$ | $v_{s}/km\cdot s^{-1}$ | $\rho/kg\cdot m^{-3}$ | $\eta/Pa\cdot s$ |
| --- | --- | --- | --- | --- | --- | --- |
| Elastic | 1 | 5 | 5.20 | 3.00 | 2500 |  |
|  | 2 | 10 | 6.10 | 3.52 | 2800 |  |
|  | 3 | 10 | 6.20 | 3.58 | 2850 |  |
| Viscoelastic | 4 | 5 | 6.00 | 3.46 | 2750 | $3.15\times{10}^{18}$ |
|  | 5 | 15 | 6.70 | 3.87 | 2950 | $3.18\times{10}^{18}$ |
|  | 6 | 15 | 7.40 | 4.27 | 3100 | $3.18\times{10}^{18}$ |
| Mantle | 7 | … | 8.00 | 4.62 | 3400 | $1.00\times{10}^{20}$ |

**REFERENCES**

1. Wu, Y. *et al.* Preliminary results pertaining to coseismic displacement and preseismic strain accumulation of the Lushan MS7.0 earthquake, as reflected by GPS surveying. *Chin. Sci. Bull.* **58,** 3460-3466 (2013).

2. Liu, X. *et al.* Preseismic deformation in the seismogenic zone of the Lushan M S7.0 earthquake detected by GPS observations. *Sci. China Earth Sci.* **58,** 1592-1601 (2015).

3. Ergintav, S. *et al.* Seven years of postseismic deformation following the 1999, M = 7.4 and M = 7.2, Izmit-Düzce, Turkey earthquake sequence. *J. Geophys. Res. Solid Earth* **114,** 1-19 (2009).

4. Goudarzi, M. A., Cocard, M. & Santerre, R. EPC: matlab software to estimate Euler pole parameters. *GPS Solut.* **18,** 153-162 (2014).

5. Li, Y.-X. *et al.* Horizontal strain field in Chinese Mainland and the surrounding areas. *Chin. J. Geophys.* **47,** 245-257 (2004).

6. Dong, D. *et al.* Spatiotemporal filtering using principal component analysis and Karhunen-Loeve expansion approaches for regional GPS network analysis. *J. Geophys. Res. Solid Earth* **111,** 1581-1600 (2006).

7. Wdowinski, S., Bock, Y., Zhang, J., Fang, P. & Genrich, J. Southern California permanent GPS geodetic array: spatial filtering of daily positions for estimating coseismic and postseismic displacements induced by the 1992 Landers earthquake. *J. Geophys. Res. Solid Earth* **102,** 18057-18070 (1997).

8. Wu, Y., Jiang, Z. & Yang, G. The application of least square collocation in obtaining information from GPS time series. *Recent Dev. World Seismol.* 99-103 (2007).

9. Jiang, Z. S. & Liu, J. N. The method for establishing the strain field and velocity field of crustal movement using least squares collocation. *Chin. J. Geophys.* **53,** 380-389 (2010).

10. Shao, Z., Wang, R., Wu, Y. & Zhang, L. P. Rapid afterslip and short-term viscoelastic relaxation following the 2008 MW7.9 Wenchuan earthquake? *Earthq. Sci.* **24,** 163-175 (2011).

**FIGURE LEGENDS**

Fig. S1 Preseismic displacement time series of each station in the east and north direction. Blue circles are original data; the green line is the data filtered by the least square collocation method; the red line is the secondary filtered data without periodicity by the least square collocation method. Map is generated by Matlab software, v. R2018a (https://www.mathworks.com/)

Fig. S2 Theoretical motion of the regional reference frame for each site in the east and north directions. Blue circles are theoretical values and the black line is the linear fitting result. The theoretical displacement in eastward direction is stable enough for transformation, while the southward component shows fluctuation. Map is generated by Matlab software, v. R2018a (https://www.mathworks.com/)

Fig. S3. Time series in the directions parallel to and normal to the seismogenic fault of the Lushan earthquake. Blue circles are original data; the green line is the data filtered by the least square collocation method; the red line is the secondary filtered data without periodicity by the least square collocation method. Map is generated by Matlab software, v. R2018a (https://www.mathworks.com/)

Fig. S4 half-space structure model10. Red and blue solid line represent Vs and Vp at each layer. Map is generated by Matlab software, v. R2018a (https://www.mathworks.com/)
